# Effects of Polyphenol Supplementations on Improving Depression, Anxiety, and Quality of Life in Patients With Depression

**DOI:** 10.3389/fpsyt.2021.765485

**Published:** 2021-11-08

**Authors:** Kelly Lin, Yanni Li, Eugene Du Toit, Lauren Wendt, Jing Sun

**Affiliations:** ^1^School of Medicine and Dentistry, Griffith University, Gold Coast, QLD, Australia; ^2^School of Pharmacy and Medical Sciences, Griffith University, Gold Coast, QLD, Australia; ^3^Menzies Health Institute of Queensland, Griffith University, Gold Coast, QLD, Australia

**Keywords:** depression, nutrition, quality of life, polyphenol, anxiety

## Abstract

**Background:** Increased prevalence of mental disorders has become a significant public health concern. Recent studies have linked nutrition to depression and anxiety, suggesting that dietary changes or nutritional supplementation may be beneficial in improving mental disorders. Polyphenols have anti-inflammatory and antioxidant properties that may counteract physiological changes in depression and anxiety. This study examined the effectiveness of polyphenol supplementation in improving depression, anxiety and quality of life (QoL).

**Methods:** Randomized controlled trials in English and with polyphenol supplementation as the intervention were searched. The primary outcome was depression, and secondary outcomes were anxiety and QoL. Only studies of at least moderate quality based on the Physiotherapy Evidence Database tool were included. Comprehensive systematic review and meta-analysis were then used to determine the effect of polyphenol supplementations on improving depression, anxiety and quality of life (QoL) in patients with depression.

**Results:** Nineteen studies with 1,523 participants were included; 18 studies (*n* = 1,523) were included in the depression meta-analysis, and 5 (*n* = 188) and 6 (*n* = 391) in the QoL and anxiety meta-analyses, respectively. Twelve of the 18 studies found significant improvements in depression with polyphenol use, while the meta-analyses results also indicated that polyphenol supplementation significantly improved depression score as compared to control conditions (MD: −2.280, 95% CI: −1.759, −0.133, *I*^2^ = 99.465). Although subgroup analyses were conducted a significantly high heterogeneity was still found amongst subgroups. Only 2 of the 5 studies found significant improvements in QoL following polyphenol supplementation and meta-analyses found that polyphenol use did not benefit QoL (MD: −1.344, *p* < 0.05, *I*^2^ = 55.763). For anxiety, 5 of the 6 studies found significant reductions in depression score following polyphenol use but meta-analyses found no significant differences in anxiety score (MD: −0.705, CI: −1.897, 0.487, *I*^2^ = 84.06) between polyphenol supplementation and control.

**Conclusion:** The results suggest that polyphenol supplementation is effective in improving depression. Physical illness may act as a risk factor that worsens depression, suggesting the need for preventative supplementation to improve depression. Polyphenol types may have varying effects, which suggests that different populations with depression may benefit from different polyphenols.

## Introduction

Major depressive disorder is a mood disorder characterized by depressed mood, anhedonia and altered cognitive function (1), and affects both psychological and physical functioning. Globally, depression has been identified as the leading cause of disability by the World Health Organization, and has been predicted to be the leading cause of disease burden by 2030 (2). Depression is a common comorbidity diagnosed among patients with chronic disease (3). In addition to depression, anxiety is another common mood disorder that affects patients diagnosed with physical illness because of increased concern about the implications of the diagnosis and stress associated with future disease management and lifestyle changes (3). In patients with chronic diseases, mood changes associated with comorbid depression and anxiety create barriers to achieving treatment adherence, and lifestyle changes critical to the management of disease (4), increasing disease severity and mortality rates (5).

Anxiety and depression are associated with poor physical and psychological well-being, which refers to quality of life (QoL) (6). QoL captures how a person perceives their overall health using a self-administered questionnaire that examines both physical and psychological well-being. This acts as an important outcome measure in patients with mental illness as it allows clinicians to understand the impact of the mental illness on patients' everyday life. Patients with comorbid depression have worse QoL, with depressed patients reporting poorer physical and psychological functioning compared with non-depressed patients (5). In postmenopausal women, depression can also significantly reduce QoL because of social impairment and cognitive dysfunction (7). Further, higher physical and psychological QoL score and overall health-related QoL have been associated with lower mortality risk (8), emphasizing the influence of mental illness on patients' perceived health and revealing the importance of QoL as a measure of patients' overall health.

Although there are various etiologies and factors that contribute to the onset of depression and anxiety, the pathophysiology of both mental illnesses is similar. Depression over-activates stress pathways (9), increases oxidative stress (10) and elevates inflammatory markers (11), resulting in altered neurotransmission, brain structural changes, mitochondrial dysfunction and neuron atrophy, which precipitate depressive symptoms (12). In a similar manner, anxiety is often triggered by overwhelming stress that results in increased oxidative stress (13) and heightened inflammatory state (14). Currently, a wide range of treatments are available for depressed and anxious patients, each targeting different etiologies. In recent years, links between nutrition and mental health have been found. Specifically, diets or supplements that are high in antioxidants and anti-inflammatories may be potentially therapeutic. A past randomized controlled trial (RCT) indicated that 6 weeks of antioxidant supplementation significantly increased plasma antioxidant levels in depressed individuals, which correlated with significantly reduced depression symptoms (15). Among the many different types of antioxidant supplementation available, polyphenols emerge as a class of antioxidant that provides a range of physiological benefits that help counteract both mental illnesses (16).

Polyphenols are natural compounds found in many plant products and are categorized into four classes: phenolic acids, flavonoids, stilbenes and lignans (17). As antioxidant agents, polyphenols exert their protective effects by upregulating the body's antioxidant reserve to reduce oxidative damage (18). Polyphenols also have anti-inflammatory properties that counteract increased inflammation in the pathophysiology of depression and anxiety. Other benefits of polyphenols include their ability to modulate cellular signaling pathways to provide neuro-protective effects and to reverse cognitive and behavioral deficits (18). Prior meta-analyses conducted on various plant extracts with polyphenols as the active nutrient have revealed significant improvements in depression associated with polyphenol supplement intake (19, 20). The effectiveness of polyphenol supplementation on improving mood and physical symptoms in specific populations, including menopausal women (21) and obese patients (22) have been indicated previously. However, the effectiveness of polyphenols in improving depression, QoL and anxiety across a range of populations is not known. Quality of life is an outcome that represents both physical and psychological well-being, while depression and anxiety are two of the most common mental disorders contributing to disabilities globally (23). These beneficial findings are limited to specific types of plant extract or diet (16), and no studies have investigated the effects of polyphenol supplementation and different types of polyphenol on depression. Thus, this meta-analysis aimed to address the gaps in current literature regarding the effectiveness of polyphenol supplementation in reduction of depression and anxiety symptoms and improvement of QoL.

## Methods

This systematic review and meta-analyses followed the Preferred Reporting Items for Systematic Review and Meta-analyses (PRISMA) approach (24). The methodology utilized has also been registered with the International Prospective Register of Systematic Reviews (https://www.crd.york.ac.uk/prospero/, Registration ID: CRD42020200069).

### Literature Search Strategy

The Population, Intervention, Control, Outcome (PICO) principle was utilized to search and identify relevant studies for this review. Two independent reviewers conducted advanced searches in PubMed, Scopus, Embase and Cochrane. The following keywords were used to identify relevant studies through advanced searches: (polyphenol OR phenolic acids OR flavonoids OR stilbenes OR lignans) AND (depression OR major depressive disorder) AND controlled studies. Reference lists of meta-analyses and systematic reviews published in the same field of research were also searched to identify additional studies. The literature search was not confined to any time frame to ensure as many relevant studies were included as possible.

### Inclusion and Exclusion Criteria

The inclusion criteria for eligible studies in this meta-analysis were:

1. Participants were over 18 years in age.
2. Participants were diagnosed with depression by using an established diagnostic tool or diagnostic inventory or diagnostic scale.
3. The study design was an RCT. The intervention used was polyphenol supplementation including four main types of polyphenol (phenolic acids or flavonoids or stilbenes or lignans). The control conditions included placebo supplementation.
4. The outcome measures were depression, QoL and anxiety.

The exclusion criteria for the studies in this meta-analysis were:

1. Depression was only measured at follow-up.
2. There were insufficient data reported for analysis.
3. The results were only reported in graphs.
4. No control group was used.

Polyphenol supplementation is the only intervention examined to elucidate the effectiveness of polyphenol on depression, anxiety and QoL as compared to control conditions. Only adults over the age of 18 were included in this analysis as adult patients and children often have different presentations and respond to treatment differently and the difference in adult participants and children would contribute to further heterogeneity. Therefore, only adults over the age of 18 were included in this study.

### Literature Screening and Data Extraction

We searched articles published between January 2000 to August 2021. All articles identified from the initial search were screened by two authors according to the Preferred Reporting Items for Systematic Reviews and Meta-Analysis (PRISMA) guidelines. After duplicate studies were disregarded, two authors independently examined the eligibility for each study by reading the title and abstract to first identify any potentially relevant studies. Any discrepancies were resolved through discussion with a third author. The full text of relevant studies identified was then assessed against the inclusion and exclusion criteria.

Data were then extracted from studies that met the eligibility criteria by using a data-collection form. The data extracted were characteristics of the study and its participants, and included study design; study population; sample size; type of polyphenol supplementation; frequency, route and duration of polyphenol supplementation; and type of depression scale used. Outcomes measured from selected studies were also recorded. For studies with incomplete data, the authors were contacted. However, no responses were received.

### Quality Assessment

Quality assessment was conducted on eligible studies using the Physiotherapy Evidence Database (PEDro) scale to assess their methodology. The PEDro scale includes 10 criteria: random allocation of subjects; allocation concealment; similarity at baseline between groups; attrition rate; blinding to subjects, assessors and researchers; use of “intention to treat” analysis; use of variability measures; and use of between-group comparison. Studies are classified into three levels according to quality: high quality (8 points or more), moderate quality (4–7 points) and low quality (3 points or less). Only studies of at least moderate quality were included in this study, and all studies included in this review scored at least 6 points on the PEDro scale.

### Statistical Analysis

The extracted data of the included studies were analyzed using the Comprehensive Meta-Analysis software version 3. A separate meta-analysis was conducted for each outcome (depression, anxiety and QoL). The overall effect size was described using random-effects meta-analyses, while standardized mean difference and 95% confidence interval were determined for each outcome. Both pooled effect size and standardized mean difference were used to measure the effectiveness of polyphenol on depression, anxiety and QoL. *I*^2^ statistics of heterogeneity were used to examine sample heterogeneity between studies. An *I*^2^ >50% or a *p* < 0.05 indicated significant heterogeneity.

Subgroup analyses were also conducted using data extracted regarding study and participant characteristics to identify potential mediating factors that might have influenced study outcomes. The factors examined for subgroup analysis included patient health status (healthy or has other pre-existing comorbidities), type of polyphenol supplementation used (isoflavone, hypericum extract or other), dose (<300, ≥300 mg), attrition rate (≥15, <15%), supplement form (tablet, capsule, or other), duration of supplementation (≤ 3, >3 months), frequency (once or more than once a day) and type of depression scale used (self-rated or clinician-rated). Because of the lack of studies that measured anxiety and QoL outcomes, subgroup analyses were only conducted for depression.

For assessment of publication bias, Egger's regression analysis and visualization of funnel plots were used. In Egger's regression analysis, *p* < 0.05 indicates significant publication bias, while *p* ≥ 0.05 suggests no significant publication bias. In addition, an asymmetrical funnel plot indicates possible publication bias, while a symmetrical funnel plot suggests no significant publication bias.

## Results

### Search Results—PRISMA

**Figure 1:**
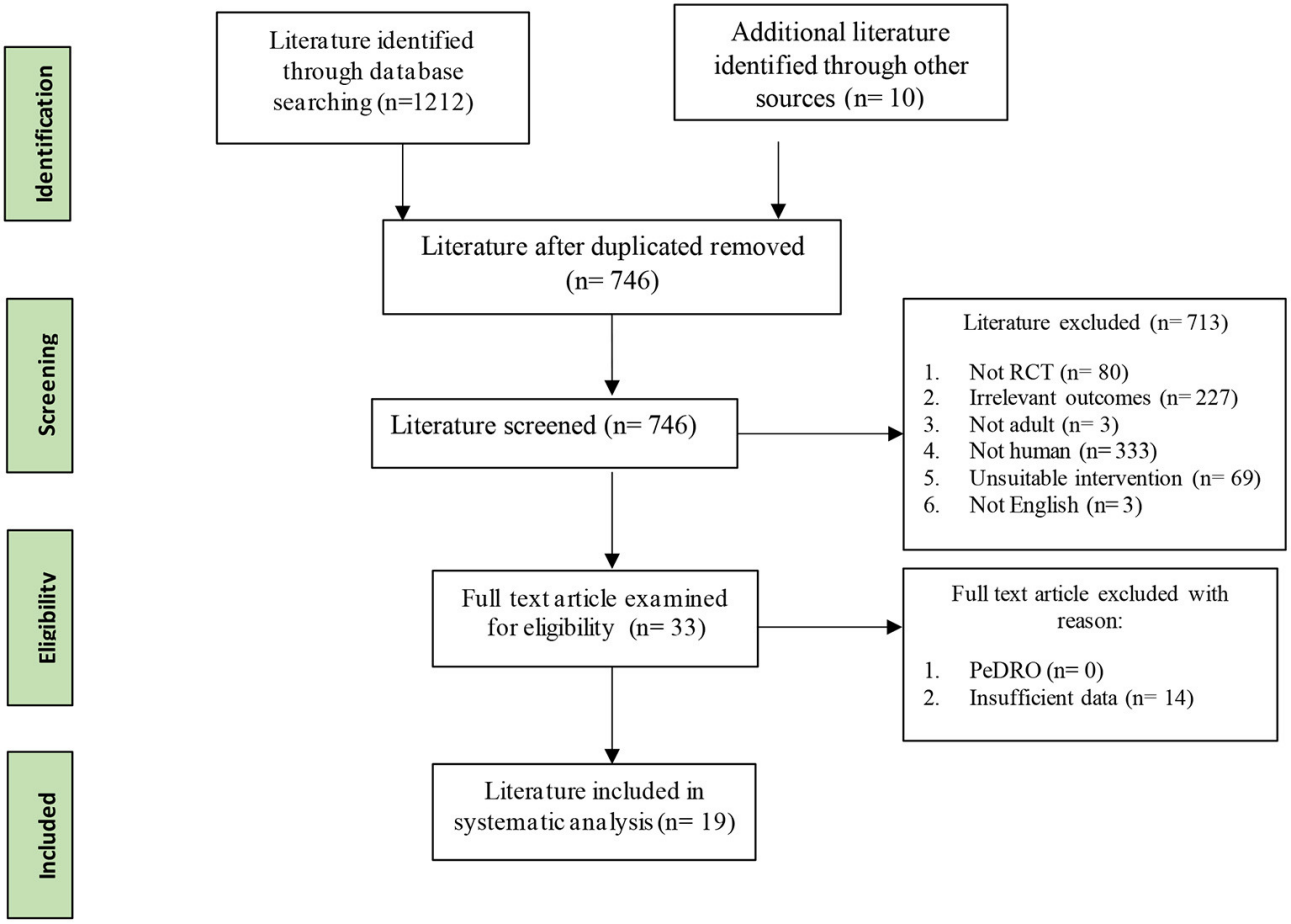
PRISMA flowchart.

As displayed in the PRISMA flowchart (Figure 1), 1,222 studies were identified in the initial search by two assessors. All identified articles were imported into EndNote, and 476 duplicates were removed. The remaining 746 studies were then screened for relevance by using titles and abstracts. Of these, 713 did not fulfill the inclusion criteria and were excluded (227 measured irrelevant outcomes; 69 used irrelevant interventions; 333 were not human studies; 3 included non-adult participants; 80 were not RCTs; 1 was not in English). Data were then extracted from the remaining 33 studies that met the inclusion criteria. However, insufficient data were reported from 15 studies. Thus, 19 studies were included in the final analysis. All included studies were of at least of moderate quality on the Pedro scale, scoring above 6 (see Supplementary Table 1).

### Characteristics of Included Studies

The detailed characteristics of included studies are displayed in Table 1. A total of 1,523 participants were included in the 19 RCTs. These studies were performed across primary, secondary and tertiary settings in Israel, Germany, Spain, Australia, Brazil, Iran, Norway, Austria, the United States, Taiwan and Korea. Eighteen studies included were analyzed for depression, while five were analyzed for QoL and six for anxiety. Of the 19 studies included, eight were designed for patients with other pre-existing health conditions and 11 were designed for participants who were only diagnosed with depression and no other comorbidities. In terms of type of polyphenol supplementation used, six studies used isoflavone, four used hypericum and eight used other types of polyphenol supplementation.

**Table 1:** Summary of studies included in meta-analysis.

						**Intervention**				**Control**					
**References**	**Design & location**	**Duration of treatment (months)**	**Route of administration**	**Depression/** **anxiety/** **QoL scale used**	**Population**	**Participants** **(n)**	**Mean** **age (SD)**	**Sex** **M (F)**	**Intervention**	**Participants** **(n)**	**Mean age (SD)**	**Sex** **M (F)**	**Control**	**Key intervention outcomes standardized mean difference (intervention/** **control)**	**Quality of study**
Atteritano et al. (25)	DB, USA	24.00	Oral	Zung Self-rating depression scale/-/36-Item Short Form Survey	Osteopenic post-menopausal women	139	53.0 (2.0)	0 (139)	Genistein	39	52.0 (2.0)	0 (123)	placebo tablet (vit D and calcium carbonate)	Depression (-5/2)	9/11
Bergman et al. (26)	DB, Israel	1.25	Oral	Hamilton Depression Rating Scale, Motgomery-Asberg Depression Scale/-/-	Patients with first episode of depression	20	65.8 (10.7)	11 (9)	Curcumin	12	61.3 (15.2)	6 (14)	placebo capsule	Depression (-2/-1.5)	9/11
Bjerkenstedt et al. (27)	DB, Sweden	1.00	Oral	Hamilton Depression Rating Scale, Motgomery-Asberg Depression Scale/-/-	Patients with mild to moderate depression	52	49.1 (12.0)	11 (43)	Hypericum extract	20	51.4 (11.8)	10 (45)	placebo tablet	Depression (9.9/9.7)	10/11
Calapai et al. (28)	DB, Italy	3.00	Oral	Beck Depression Inventory, Hospital Anxiety and Depression Scale, Mini Mental State Examination/-/-	Healthy older adults	57	66.9 (5.3)	27 (30)	Cognigrape extract	60	66.9 (5.2)	26 (28)	maltodextrin	Depression (1.3/0.1)	10/11
de Sousa-Munoz and Filizola (29)	DB, Brazil	4.00	Oral	Center for Epidemiological Studies Depression/-/-	climacteric outpatients	39	-	-	Isoflavone	73	-	-	placebo tablet	Depression (-4.3/-3.6)	7/11
Firoozabadi et al. (30)	DB. Iran	1.00	Oral	Beck Depression Inventory/Beck Anxiety Inventory/-	Patients with anxiety and depression	34			N. menthoides	42	36 (9.8)	9 (27)		QoL (-11.8/-5)	11/11
Hirose et al. (31)	DB, Japan	2.00	Oral	Hospital Anxiety and Depression Scale/-/Menopausal Symptom Scale	Menopausal women	29	47.6 (4.9)	0 (29)	Isoflavone	29	48.0 (5.7)	0 (29)	Placebo tablet	Depression (-1.1/-0.3), QoL (-1.2/ 1)	8/11
Davidson et al. (32)	DB, USA	6.25	Oral	Hamilton Depression Rating Scale/-/-	Patients with major depressive disorder	113	43.1 (13.5)	40 (73)	Hypericum extract	16	40.1 (12.2)	39 (77)	Placebo	Depression (-7.8/−6.8)	8/11
Ibero-Baraibar et al. (33)	RCT, Spain	1.00	Oral	Beck Depression Inventor/Spanish translation of the State–Trait Anxiety Inventory/-	Overweight or obese adult	23	58.0 (5.6)	11 (12)	Cocoa extract	30	57.0 (5.0)	11 (13)	Lactose	Depression (-3.7/-5.7), Anxiety(-0.8/1.2)	8/11
Ishiwata et al. (34)	RCT, Japan	3.00	Oral	Profile of Mood States/Profile of Mood States/ Menopausal Symptom Scale	Menopausal women	10	50.5 (4.7)	0 (10)	Isoflavone	25	50.6 (4.9)	0 (14)	placebo capsule	Depression (1.8/ 0.1), QoL (4.9/6.2), Anxiety (-0.7/0.6)	8/11
Kanchanatawan et al. (35)	DB, Thailand	3.00	Oral	Montgomery-Asberg Depression Rating Scale/-/-	Patients with major depressive disorder	33	42.6 (13.6)	9 (27)	Curcumin	1,046	46.2 (13.4)	10 (22)	Placebo tablet	Depression (-15/-7)	8/11
Lingaerde et al. (36)	DB, Norway	2.25	Oral	Montgomery-Asberg Depression Rating Scale/-/Visual Analog Scale	Patients with seasonal affective disorder	15	40.6 (8.0)	4 (11)	Flavone	1,046	40.1 (10.5)	2 (10)	Placebo tablet	Depression (7.3/4.7)	8/11
Lipovac et al. (21)	DB, Austria	3.00	Oral	Hospital Anxiety and Depression Scale/Hospital Anxiety and Depression Scale/-	Post-menopausal women	50	54.5 (6.2)	0 (50)	Isoflavone	26	53.7 (7.8)	0 (59)	Placebo capsule	Depression (-7.4/-1.7), Anxiety (-7.6/-1.9)	9/11
Lopresti et al. (37)	DB, Australia	2.00	Oral	Inventory of Depressive Symptomatology/ Spielberger State-Trait Anxiety Inventory/-	Patients with major depressive disorder	28	44.0 (11.9)	8 (20)	Curcumin	9	48.5 (11.7)	8 (20)	Cellulose capsule	Depression (-10.3/-7.2), Anxiety (-9.7/-7.1)	10/11
Miodownik et al. (38)	DB, Isarel	6	Oral	Calgary Depression Scale/-/-	Patients with chronic schizophrenia	20	54.1 (12.9)	14 (6)	Curcumin	36	53.4 (14.9)	11 (7)	Placebo capsule	Depression (-1.4/−1.1)	8/11
Rapaport et al. (39)	RCT, USA	3	Oral	Hamilton Depression Rating Scale/-/Quality of Life Enjoyment and Satisfaction Questionnaire	Patients with minor depression	26	42.2 (14.1)	13 (13)	St. John's Wart	1,458	51.4 (16.6)	12 (11)	Placebo tablet	Depression (-5.6/-7.1), QoL (7.2/9)	10/11
Jung-Gum et al. (40)	DB, Korea	3	Oral	Beck Depression Inventory/-/Visual Analog Scale	Single Women	16	25.9 (4.8)	0 (16)	Hypericum extract	26	25.6 (4.3)	0 (14)	Lactose, cellulose	Depression (2.5/ 1.3), QoL (0.1/ 0.4)	7/11
Santos- Galduróz et al. (41)	DB, Brazil	4.00	Oral	Geriatric Depression Scale/-/-	Menopausal Women	19	54.4 (4.3)	0 (19)	Isoflavone	94	56.6 (3.6)	0 (19)	Placebo tablet	Depression (-2.7/-5.1)	6/11
Yang et al. (42)	DB, Taiwan	6.00	Oral	Women's Heath Questionnaire/ Women's Health Questionnaire/-	Peri-menopausal Women	80	46.7 (5.1)	0 (80)	Pycnogenol	44	47.0 (4.2)	0 (75)	Placebo capsule	Depression (0.4/0), Anxiety (0.4/0.02)	8/11

### Polyphenol Supplementation

A range of polyphenols were assessed in the 19 articles included in this study. Six studies used isoflavone, 4 studies used hypericum, and 4 studies used curcumin. Other polyphenol supplementation used included genistein, cognigrape extract, cocoa extract, N. menthoides and pycnogenol. In terms of dosage, 8 studies used polyphenol supplements with a dosage <300 mg, while 11 studies used polyphenol supplements with a dosage ≥ 300 mg. A range of supplement formulation was used amongst the included studies. Nine studies administered the supplement in the form of a capsule, 7 studies provided tablet supplementation, while the remaining 3 studies administered polyphenol through ready to eat meals, pill formulation and powder. The amount of times polyphenol supplementation was administered within a day also differed. Seven studies administered supplementation once a day, participants from 9 studies took the supplement more than once a day, while 3 studies did not report the frequency of supplementation intake in a day. Another difference between studies was the duration of the supplementation period, with 13 studies having a supplementation period ≤ 3 months and 6 studies with a supplementation period >3 months.

### Polyphenol Supplementation Treatment Effects

#### Effects on Depression

The effect of polyphenol supplementation on depression was reported by 18 of the included studies with a total sample of 1,523 participants. Overall, majority of the studies included found a significant association with polyphenol use and improvement in depression scores. The effect of polyphenol on depression and other outcomes have been summarized in Table 2. Twelve of the 18 studies included for analyses found significant association improvement in depression scores after polyphenol supplementation while 6 studies found no statistically significant improvements associated with polyphenol used. Both self-rated and clinician rated depression scales were used to determine the participants depression score. Six studies used self-rated depression scales and 13 studies used clinician rated depression scales. Both studies with patient populations with and without other comorbidities were included. Ten studies had patients that were otherwise healthy (26–29, 32, 35, 36, 39–41) and 8 studies had patients diagnosed with additional comorbidities. A proportion of the studies (*n* = 6) examined menopausal women (21, 25, 29, 31, 34, 42). Other comorbidities within patient populations included obesity (33) and schizophrenia (38). Some of the studies found significant improvements in systematic symptoms such as fatigue and insomnia (29, 31). Other studies have also identified improved somatic symptoms, especially amongst post-menopausal populations.

**Table 2:** Standardized mean difference and effect size of polyphenol intervention on depression, anxiety and QoL.

			**Mean difference**			**Effect size**		
**Variables**	**Studies (n)**	**Participants**	**MD (95% CI)**	**I^2^ (%)**	**Q-test**	**Effect size (95% CI)**	**I^2^ (%)**	**Q-test**
Depression	18	1,523	−2.280 (−1.759, −0.133)*	99.465*	3179.05	−0.1562 (−1.076, 0.758)	98.128*	907.925
Anxiety	6	461	−0.705 (−1.8978, 0.487)	84.06***	37.641	−0.217 (−0.777, 0.342)	82.454***	34.197
QoL	5	188	−1.344 (−2.300, −0.387)*	55.763	9.042	−0.530 (−1.077, 0.017)	68.980*	12.895

*p ≤ 0.05, ***p ≤ 0.001.

*QoL, Quality of life*.

**Figure 2:**
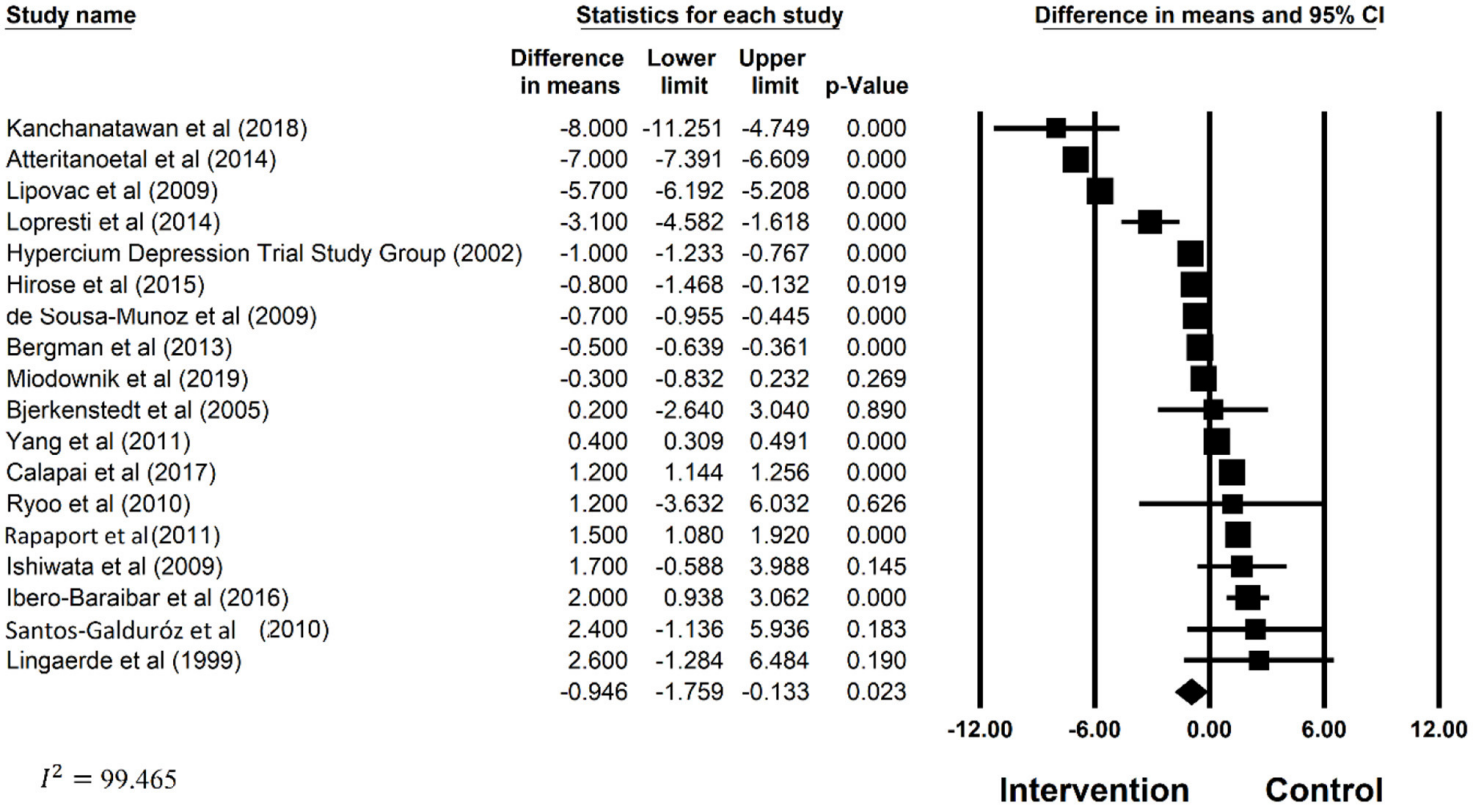
Forest plots of the effect polyphenol supplementation on depression.

The estimated mean difference in depression score was −2.280 (CI: −1.759, −0.133), which suggested significant improvements in depression associated with polyphenol supplementations. Significant heterogeneity between the studies included (*I*^2^ = 99.465%, *p* = < 0.05) were noted. Forest plots for the effect of polyphenol on depression has also been provided in Figure 2. Subgroup analysis on patient health status, type of polyphenol supplementation used, dosage, attrition rate, supplement form, duration of other supplementation, frequency and type of depression scale was examined. The results of subgroup analysis has been displayed in Table 3. Polyphenol supplementation were significantly more effective in improving depression in patients with other pre-existing comorbidities (Effect Size: −0.652, *p* < 0.001) as compared to depressed patients without other pre-existing comorbidities. In terms of the type of polyphenol supplementation used, isoflavone (Effect Size: −0.891, *p* < 0.001) was most effective in improving depression followed by other types of polyphenol (Effect Size: −0.499, *p* < 0.001) and lastly, hypercium extract (Effect Size: −0.398, *p* < 0.001). Additionally, polyphenol supplements were most effective in reducing depression when supplements were administered in capsule form (Effect Size: −0.925, *p* < 0.05), followed by other forms (Effect Size: 0.676, *p* < 0.001), then tablet form (Effect Size: −0.339, *p* < 0.001). Subgroup analysis also found that polyphenol supplementation was more effective in improving depression in studies with the following study characteristics: attrition rate <15%, study duration >3 months, studies with polyphenol dosage <300 mg, and studies where a clinician rated depression scale was used. Despite the stratification of several significant mediating factors, the heterogeneity still remained high for the studies within each subgroup.

**Table 3:** Subgroup analysis on the effect of polyphenol on depression.

	**Effect size**					
**Subgroups**	**Studies (n)**	**Participants**	**I^2^ (%)**	**Q-test**	**Effect size (95% CI)**	***P*-value**
**Health status**						
Pre-existing condition	8	731	98.746	558.026	−0.652 (−0.841, −0.463)	<0.001
Healthy	10	792	97.414	348.075	−0.481 (−0.641, −0.321)	<0.001
**Drop out**						
>/= 15%	4	463	98.053	154.110	0.061 (−0.138, 0.261)	0.548
<15%	14	1,060	98.132	695.755	−0.921 (−1.075, −0.766)	<0.001
**Duration**						
< /= 3 months	10	798	97.629	463.938	−0.218 (−0.395, −0.040)	0.016
> 3months	8	725	98.803	417.870	−0.857 (−1.025, −0.687)	<0.001
**Form**						
Tablet	8	568	98.573	490.402	−0.339 (−0.535, −0.143)	0.0007
Capsule	7	745	98.314	355.773	−0.925 (−1.094, −0.755)	<0.001
Others	3	210	43.198	3.521	0.676 (0.270, 1.082)	0.001
**Type of supplement**						
Isoflavone	6	332	96.465	141.457	−0.891 (−1.145, −0.636)	<0.001
Hypercium extract/SJW	4	417	96.228	79.534	−0.398 (−0.602, −0.194)	<0.001
Others	8	774	98.967	677.651	−0.499 (−0.689, −0.308)	<0.001
**Treatment frequency**						
Once a day	6	432	98.058	257.523	−0.168 (−0.394, 0.057)	0.144
More than once a day	9	671	95.238	168.351	−0.178 (−0.340, −0.015)	0.032
Others/not reported	3	420	99.212	253.761	−2.902 (−3.230, −2.574)	<0.001
**Dosage**						
<300 mg	8	705	98.830	598.154	−1.380 (−1.597, −1.163)	<0.001
>/= 300 mg	10	818	96.050	227.822	−0.167 (−0.315, −0.019)	0.027
**Type of depression scale used**						
Self-rating		799	98.811	672.661	−0.262 (−0.445, −0.076)	0.005
Clinician Rated		724	96.238	217.847	−0.786 (−0.950, −0.621)	<0.001

#### Effects on Anxiety

Six articles (*n* = 461) included in this study measured the effects of polyphenol supplementation on anxiety scores. The majority of the studies (*n* = 5) found a statistically significant improvement with anxiety score following the use of polyphenol supplementation. One study (21) found a significant improvement in anxiety score by 76% following polyphenol supplementation as compared to a 21.7% improvement in the placebo group. Another study also found significant improvements in anxiety throughout the 6 months supplementation period (42). The anxiety score improved from baseline of 2.85–3.27 after 3 months of supplementation and remained at 3.27 following 6 months of supplementation, while the placebo group had no significant improvements in anxiety score at all time points.

Two of the studies included post-menopausal and peri-menopausal female participants and thus utilized scales specific to women including the Menopausal symptom scale and the Women Health Questionnaire (WHQ) to measure anxiety score (34, 42). Two other studies both used State-Trait Anxiety Inventory (STAI) (37), with one of the study using a validated Spanish translation version for the Spanish population (33). Lastly, one study used the Hospital Anxiety and Depression Scale (HADS) to assess anxiety (31), and another study using the Beck Anxiety Inventory (BAI) (30). Five of the 6 studies also measured depression.

The mean difference in anxiety score between supplementation group and control was −0.705 (CI: −1.897, 0.487) with significant level of heterogeneity (*I*^2^ = 84.06, *p* <0.05) No further subgroup analysis could be conducted due to the lack of studies in each subgroup.

#### Effects on Quality of Life

To determine the effectiveness of polyphenol supplementation on QoL, 5 studies with a population size of 188 were included. In comparison to depression and anxiety outcomes, less than half (*n* = 2) of the studies reported a statistically significant association between polyphenol supplementation and improved QoL. Both physical and psychological aspects of QoL was measured across the studies included. Two studies used visual analog scales (VAS) to measure a range of QoL related factors including energy level, tiredness, appetite, and depression (36, 40). One study used the Menopausal Symptom Scale (MSS) to measure physical symptoms and mood (34). Another study used Quality of Life Enjoyment & Satisfaction questionnaire (Q-LES-Q) (39), and one study used Athens Insomnia Scale (AIS) (31). All five studies included for QoL assessment also measured depression and one of the studies also reported anxiety results. One study found significant improvements in somatic and vasomotor symptoms associated with menopause following polyphenol supplementation (34). Another study also found significant improvements in somatic symptoms in menopausal women, as well as improved insomnia (34). Overall, only studies with populations of menopausal women found significant improvements in Qol (31, 34). In contrast, in a study with a population of women with an average age of 25.7, no significant improvements in premenstrual syndrome symptoms including fatigue or mood were found (40). Other 2 studies that included participants without other comorbidities also found no significant improvements in QoL (36, 39, 40).

A significant mean difference of −1.344 (95% CI: −2.300, −0.387) in QoL score was estimated, indicating that polyphenol supplementation did not result in an improvement in Qol when compared to placebo. Although statistically insignificant, a significant level of heterogeneity (*I*^2^ = 55.763, *p* = 0.060) was found. No further subgroup analysis was conducted due to the lack of studies in each subgroup.

### Publication Bias

The results of publication bias assessment has been presented in Table 4. Egger's regression analysis indicated that no significant publication bias was found for all outcomes—depression, anxiety and QoL. The funnel plots for all outcomes were symmetrically dispersed, further indicating minimal publication bias.

**Table 4:** Egger's regression analysis on publication bias of included studies.

**Outcomes**	**Studies(n)**	***T*-value**	**95% CI**	***P*-value**
Depression	18	2.095	−15.265, 0.090	0.052
QoL	5	0.300	−8.063, 6.491	0.779
Anxiety	6	2.141	−31.528, 6.171	0.122

## Discussion

### Effects on Depression

In line with prior studies, this meta-analysis found that polyphenol supplementation significantly reduced depression score compared with the control. A past meta-analysis found significant associations between consumption of Mediterranean diets high in polyphenols and improved depression score and reduced depression symptoms (16). Many included studies tested the effectiveness of polyphenol supplementation on depressed individuals with other comorbidities. Previous studies as well as studies included in this review found significant improvements in somatic symptoms that contributes to depression (34, 43). The reduced severity of physical symptoms may help explain improved depression in populations with additional comorbidities reviewed in this study. Those with pre-existing conditions and chronic illnesses are often associated with worsened depression outcomes and symptoms (44, 45). Progression of depression also positively correlated with progression of chronic disease (45). The worsened depression state in patients with chronic conditions may be explained by the effects of somatic symptoms including chronic fatigue and pain linked with their physical illnesses (3). This suggest that physical illness and especially chronic conditions may pose as a threat on patient's mental health due to increased stress from the diagnosis, management, and economic burden of disease (44). Thus, participants with pre-existing conditions may have a greater depression score due to the impact of their physical illness on their mental health. Patients with pre-existing conditions may also benefit from polyphenol supplementations more than those without pre-existing conditions.

Among participants with pre-existing conditions in this meta-analysis, a notable proportion were from studies with postmenopausal women. Depression in this population is often associated with increased oxidative stress linked to reduced estrogen levels (46) due to loss of ovarian and follicle development after menopause. Estrogen has antioxidant properties, so the lowered estrogen levels increase the risk of various physical illnesses including cardiovascular disease, stroke and depression. Polyphenols have antioxidant properties that can counteract the elevated oxidative stress following menopause. Prior studies have also demonstrated that polyphenols' antioxidant properties may help improve depression through targeting neuronal survival, regeneration and development (47). Further, as a lipid-rich organ, the brain is highly susceptible to oxidative damage (48), meaning that improved antioxidant status from polyphenol supplementation may further aid in countering depression-induced disturbances in neurotransmission.

Thus, with polyphenol's potent antioxidant and anti-inflammatory properties, all types of polyphenols analyzed in this study resulted in a significant improvement in depression score. Although all supplements included in this review are classified as polyphenols, each supplement have additional benefits that may improve depressive mood in different ways. Isoflavone is considered as phytoestrogens that is often used a supplement post-menopause as they exert estrogen-like effects that decline after menopause. In line with this study, previous meta-analyses have indicated that phytoestrogens with isoflavone as the active ingredient had a significant positive effect in postmenopausal women with depression and anxiety disorders (21, 49). The additional estrogen-like effects that isoflavone has may improve depression as estrogen interacts with dopaminergic, serotonergic, and cholinergic systems, and brain regions involved in the control of mood and higher cognitive functions (50). Thus, the additional benefits of isoflavone as a phytoestrogen may explain why isoflavone appears to be the most effective polyphenol in improving depression. Nonetheless, this review demonstrated that polyphenols supplementation may improve depression regardless of the type of polyphenol supplemented.

A variety of depression scales were also used amongst the studies included for review, which may have affected the results. Past studies have also showed varying results regarding self-assessed and clinician assessed depression scores. One study indicated moderate to strong correlations between self-reported questionnaires and clinician-rated depression scales (51), while another indicated that self-reported and clinician-assessed depression scores are not equivalent (52). Despite the discrepancies, both methods have been recognized as a valid measure of depression in clinical studies. One study demonstrated a slight advantage with the use of clinician-rated scales and hypothesized that clinician-rated depressions scales may be more sensitive to change (53). However, because of the small number of papers collected for this study, further study needs to focus on more objective and sensitive measures in depression. Further, because of differences in results, some investigators have suggested the need to include both measures for a more complete assessment (52).

### Effects on Anxiety

Although 5 of the 6 studies included identified a significant improvement in anxiety score following polyphenol supplementation, no significant improvements were found through meta-analyses. This differed from previous studies that found previous meta-analyses have indicated that phytoestrogens with isoflavone as the active ingredient had a significant positive effect in postmenopausal women with depression and anxiety disorders (21). The difference in results may be due to the similarity between anxiety and depression symptoms, making it difficult to distinguish especially when utilizing a self-rating scale. The use of self-administered questionnaires to assess depression and anxiety may not be as sensitive as clinician-rated scales (53), and may only measure general psychological distress (54). Moreover, patients experiencing psychological distress are often comorbid with both depression and anxiety, further complicating the assessment process (55).

### Effects on Quality of Life

Two of the 5 studies included in the meta-analysis found significant improvements in QoL. However, the overall meta-analyses result found that polyphenol supplementation was not associated with improved overall QoL score. The 2 studies that found a significant association both examined the effects of polyphenol on women with menopause (31, 34), suggesting that the beneficial effect of polyphenol on QoL may be limited to menopausal women. Both studies found significant improvements in various aspects of menopausal symptoms, including significantly reduced depression, anxiety and somatic symptoms.

The significant improvements in physical functioning and mental health in populations of menopausal women may be associated with the beneficial antioxidant effects of polyphenol. Following menopause, oxidative stress increases with reduced estrogen levels (46). Polyphenols have antioxidant properties that can target depression through targeting neuronal survival, regeneration and development (47) and physical functioning by reducing fatigue and pain (33, 56). Thus, polyphenol supplementation may potentially target both impacted mental health and physical functioning in menopause to result in an improvement in overall QoL.

In contrast, 3 studies included in the analyses found no significant improvement in QoL. Although meta-analyses in this study found significant mean difference in QoL following polyphenol use, the significant difference found did not favor polyphenol use. The non-beneficial effect found here may be due to the lack of studies included for QoL analysis. Of the 18 studies included for depression meta-analyses only 5 measured QoL outcomes, which reduced the power of the meta-analyses results for QoL. To determine the effects of polyphenol on QoL, more studies must be included in future meta-analyses. Effects on physical functioning could also be analyzed in addition to mental health to elucidate the overall effect of polyphenol on QoL.

### Limitations

This study included 19 RCTs that used polyphenol supplementation as an intervention for depression or anxiety treatment and yielded high quality results. However, one significant limitation is the high level of significant heterogeneity found amongst the studies included. Despite the conduction of subgroup analysis, the heterogeneity remained high within subgroups stratified. The high level of heterogeneity may be explained by the varied dosage, forms of polyphenol supplementation and duration of intervention use. Another limitation is the disproportionately high number of females included in the analysis, as many of the studies examined the use of polyphenol supplementation in post-menopause women. The population examined in this study also only included adult participants over the age of 18, which limits the applicability of the results. Lastly, the results of this study are limited to the short term effects of polyphenol supplementation as the supplementation were only administered for <4 months in most studies. Thus, given these limitations, the results of this study must be read and interpreted with caution.

## Conclusion

Findings from this meta-analyses and systematic review have demonstrated the effectiveness of a range of polyphenol supplementation on reducing depression level and improving anxiety. A range of different polyphenol supplementations was investigated across the current literature and both participants with and without additional comorbidities other than depression have also been studied. This review found that a great proportion of the included studies had female participants only and aimed to investigate the effectiveness of polyphenol supplementation on psychological symptoms surrounding menopause. Thus, a gap in literature exists between the effectiveness of polyphenols on men. Meta-analyses results found no significant association between polyphenol use and anxiety, while polyphenol use did not improve QoL. Nevertheless, all types of polyphenol supplementation analyzed in this study produced a significant improvement in depression score, providing substantial evidence that supports the links between use of polyphenol supplementation and mental disorders. The significant improvement in depression further supports the addition of polyphenol as an adjunct nutrient to complement current treatment regimes.

## Data Availability Statement

The original contributions presented in the study are included in the article/Supplementary Material, further inquiries can be directed to the corresponding author/s.

## Author Contributions

JS has contributed to study design, conceptualization of the study and study design, supervision of the study team, analyzed data, and wrote up of the manuscript. KL contributed to the literature search, data extraction, and draft write up. YL contributed to the literature search. ET and LW contributed to the critical review of the manuscript. All authors contributed to the article and approved the submitted version.

## Conflict of Interest

The authors declare that the research was conducted in the absence of any commercial or financial relationships that could be construed as a potential conflict of interest.

## Publisher's Note

All claims expressed in this article are solely those of the authors and do not necessarily represent those of their affiliated organizations, or those of the publisher, the editors and the reviewers. Any product that may be evaluated in this article, or claim that may be made by its manufacturer, is not guaranteed or endorsed by the publisher.

## Abbreviations

PEDro: Physiotherapy Evidence Database
PICO: Population, Intervention, Control, Outcome
PRISMA: Preferred Reporting Items for Systematic Reviews and Meta-Analysis
QoL: quality of life
RCT: randomized controlled trial.
