# Supplementary material for: Effects of Polyphenol Supplementations on Improving Depression, Anxiety, and Quality of Life in Patients With Depression
Source: Front Psychiatry. 2021 Nov 8;12:765485. doi: 10.3389/fpsyt.2021.765485 (PMC8606635; doi:10.3389/fpsyt.2021.765485)
Supplement: Supplementary file 1 [file Table_1.docx]

**Supplementary Table 1: Quality assessment of included studies using Pedro Scale**

| **Study** | **Specified Eligibility Criteria** | **Random Allocation** | **Allocation Concealment** | **Blinding of Subjects** | **Blinding of Assessors** | **Blinding of Researchers** | **Similar at baseline** | **Key outcome measurement 85%** | **Intention to treat** | **Between group comparison** | **Measures of variability** | **Total score** |
| --- | --- | --- | --- | --- | --- | --- | --- | --- | --- | --- | --- | --- |
| **Atteritanoetal et al (2014)** | **X** | **X** | **-** | **X** | **-** | **X** | **X** | **X** | **X** | **X** | **X** | **9/11** |
| **Bergman et al (2013)** | **X** | **X** | **X** | **X** | **X** | **-** | **X** | **X** | **-** | **X** | **X** | **9/11** |
| **Bjerkenstedt et al (2005)** | **X** | **X** | **X** | **X** | **X** | **X** | **X** | **-** | **X** | **X** | **X** | **10/11** |
| **Calapai et al (2017)** | **X** | **X** | **X** | **X** | **X** | **-** | **X** | **X** | **X** | **X** | **X** | **10/11** |
| **de Sousa-Munoz et al (2009)** | **X** | **X** | **-** | **X** | **X** | **-** | **-** | **X** | **-** | **X** | **X** | **7/11** |
| **Firoozabadi et al (2016)** | **X** | **X** | **X** | **X** | **X** | **X** | **X** | **X** | **X** | **X** | **X** | **11/11** |
| **Hirose et al (2015)** | **-** | **X** | **X** | **X** | **X** | **-** | **X** | **X** | **-** | **X** | **X** | **8/11** |
| **Hypercium Depression Trial Study Group (2002)** | **X** | **X** | **X** | **X** | **X** | **-** | **X** | **-** | **-** | **X** | **X** | **8/11** |
| **Ibero-Baraibar et al (2016)** | **X** | **X** | **-** | **X** | **X** | **-** | **X** | **X** | **-** | **X** | **X** | **8/11** |
| **Ishiwata et al (2009)** | **-** | **X** | **-** | **X** | **X** | **X** | **X** | **-** | **-** | **X** | **X** | **8/11** |
| **Kanchanatawan et al (2018)** | **X** | **X** | **-** | **X** | **X** | **-** | **X** | **-** | **X** | **X** | **X** | **8/11** |
| **Lingaerde et al (1999)** | **X** | **X** | **X** | **X** | **X** | **-** | **X** | **-** | **-** | **X** | **X** | **8/11** |
| **Lipovac et al (2009)** | **X** | **X** | **X** | **X** | **X** | **-** | **X** | **-** | **X** | **X** | **X** | **9/11** |
| **Lopresti et al (2014)** | **X** | **X** | **X** | **X** | **X** | **-** | **X** | **X** | **X** | **X** | **X** | **10/11** |
| **Miodownik et al (2019)** | **X** | **X** | **-** | **X** | **X** | **-** | **X** | **X** | **-** | **X** | **X** | **8/11** |
| **Rapaport et al (2011)** | **X** | **X** | **X** | **X** | **X** | **X** | **X** | **-** | **X** | **X** | **X** | **10/11** |
| **Ryoo et al (2010)** | **X** | **X** | **-** | **X** | **X** | **-** | **X** | **-** | **-** | **X** | **X** | **7/11** |
| **Santos-Galduróz et al (2010)** | **X** | **X** | **-** | **X** | **X** | **-** | **-** | **-** | **-** | **X** | **X** | **6/11** |
| **Yang et al (2011)** | **X** | **X** | **X** | **X** | **X** | **-** | **X** | **-** | **-** | **X** | **X** | **8/11** |
